# Homæopathy, Allopathy, and “Young Physic”

**Published:** 1846-07

**Authors:** John Forbes

**Affiliations:** Editor of the British and Foreign Medical Review, etc.


ART. VI.—*Homœopathy, Allopathy, and "Young Physic."* By John Forbes, F. R. S., *Editor of the British and Foreign Medical Review, etc.*

An article bearing the above title, which originally appeared in the British and Foreign Medical Review, from the pen of the editor of that Journal, has been reprinted in a separate form, and republished in this country by Lindsay and Blackiston, of Philadelphia. We have not received the original article, and our general knowledge of its character is derived from notices and abstracts which we have met with in our exchange Journals. Dr. Forbes, it appears, has discussed the practical claims of Homœopathy with a more than lenient spirit. Assuming (what every person competent to entertain a rational opinion on the subject must of course, admit) that the system, as a system of therapeutics, is purely negative, and that all its positive influences are exerted exclusively through the mind of the patient, he appears willing to concede, or rather he candidly states that its results may in some instances compare favorably with the average of regular practice. The inference to be drawn from this concession is, that medical practice, in the aggregate of its results, may be less beneficial than is sometimes supposed, and that, at all events, the necessity of a reformation exists, which reformation it belongs to the active, enthusiastic spirits of the present age, which he personifies under the title of "Young Physic", to carry out.

Admitting the premises, this deduction would certainly appear to be legitimate. Not having read the entire article, we are not able to review the facts upon which the premises are based; but without professing to have bestowed deliberate investigation upon the subject, or to be in possession of data for proper discussion of the proposition, we are free to confess to the probability that the conclusion is correct. If the question "is practical medicine productive of a greater amount of harm than good to mankind," be fairly put, we have no reluctance in admitting that an argument can be made for the affirmative, although we do not by any means offer as our opinion that the affirmative is true. We concede that the subject is a debateable one. 'What, then,' it may be said; "do you, a practitioner of medicine, doubt the utility of Medical Science? Do you practice your profession with a kind of uncertainty whether it is doing more good than harm!" Such are the exclamations which might be made by the superficial reader. They who subsist by depreciating and slandering the medical profession, and they who have imbibed disgust and opposition from a partial experience and observation, greedily seize upon such an admission, and do not fail to make the most of its availability, the former for their nefarious projects, and the latter to confirm their sweeping generalizations. This (as might be expected) appears to have been the case with the published sentiments of Dr. Forbes. The Homœopathists quote portions of his article with triumph, and quacks of every stamp [and God knows how large a number, and how great a diversity are herein comprised] are in ecstacies at an opportunity to lay claim to an ally in so eminent a practitioner and writer as Dr. Forbes.

This was to have been expected. Any concession by a member of the profession, especially if he be a distinguished member, of imperfection in the existing state of Medical science, and of fallibility in the profession, is certain of being the occasion of misrepresentation and misapplication by those whose interests are concerned in diminishing public confidence in the science and profession. This, however, is no reason why the actual condition and resources of medical knowledge and art should not be candidly considered, but, on the other hand, it furnishes, in itself, a reason why it should be attempted to place the subject in a proper light before the public mind. There are some unfortunate errors which have a popular currency, and especially prevail among those who regard the medical profession with distrust, which it behoves medical men to endeavor to correct. One of these errors is the idea that medicine is a system of doctrines and rules, which are handed down unchanged from instructor to pupil, like a religious creed, from which it is rank heresy to swerve. This is what is meant when such terms are used as "old school," "regular system," &c. Now it is quite needless to argue that this is a very false and unjust view of the case. Certainly there is no lack of freedom of opinion, or latitude of doctrine in the medical profession. Every one is at liberty to construct his own articles of faith, and to shape his practice accordingly; and so far from there being any disposition to check this freedom, we all know that the tendency has always been directly the reverse. The public however, do not always so understand it. It is not considered that medicine is a progressive science, but it is thought to consist of formulas which every member feels bound to sustain as permanent fixtures, never being willing to admit that they are not complete, nor sanctioning the least innovation. It is by fostering this mistaken impression that the advocates of absurd doctrines like Homœopathy create an apparent issue with us, as if we were dogmatical adherents to antiquated and exclusive opinions, and they, persecuted reformers. At the same time, these very doctrines, which claim to appropriate the position occupied by medical science, invariably assume to be unchangeably perfect, leaving no room for farther improvement — being emphatically obnoxious to precisely the objections which are unjustly charged upon medicine.

Another popular error is to consider medical science, and the medical profession, one and indivisible. The two are by no means inseparable, and each, of necessity, an index of the other. So far from this, while it may be true that in its practical applications it may be susceptible of enough of abuses to be productive of as much injury as good, it does not follow that the science has not been, and is not capable of conferring inestimable service to mankind. Conceding, for the sake of illustration, that the members of the profession collectively represent the abuses, rather than the advantages of medical science, this concession does not disparage medicine, but only shows the necessity of making the distinction just referred to. When we speak of medicine, we mean the science in its present condition of progress, possessing certain capabilities for useful results. We believe these capabilities are very great. Not that all its capabilities have yet been developed; nor that they will not continue to spring up indefinitely; but they are even now vast, and mankind should be grateful for them. But how are their useful results to be effected? They are to be carried into effect through the instrumentality of numerous individuals. Here the dependence is upon something additional to the intrinsic capabilities of the science. The abilities and qualifications of the different members of the medical profession, are involved. Now, although there can be no doubt respecting the usefulness of which medical science is capable, there may be some room for doubt whether the amount of its possible usefulness is exemplified in the sum total of all that is done under the name of medical practice, even in the hands of legal or regular practitioners. The public, however, or they who cavil at the medical profession, are apt to consider the science and the profession as synonymous terms. They hold the science responsible for the doings of every member of the profession, and predicate of the former from what comes under their observation concerning the latter. It would probably appear to such persons every unreasonable to estimate the efficiency of religious truth in preventing iniquity of conduct, by the occasional derelictions of those who make professions of piety; yet the absurdity in such a case is not greater than in making medicine accountable for the ignorance, the stupidity, or the venial errors of every individual who has claims to be regularly or legally recognised as a practitioner of medicine.

If the public mind could be disabused of these, and other erroneous impressions relating to our science and profession, we cannot but think it would tend to elevate the former in a measure above the multitudinous delusions and impositions which now prevail, and secure for the latter a more rational appreciation of their value, and a more cordial co-operation for their interests. But to return to Dr. Forbes. That he is one of the last, willingly to do aught to promote, conciliate, or compromise with quackery, in any shape, we do not doubt; but in treating of Homœopathy, in so far as we may form an opinion from the extracts and comments which we have seen, it does seem that he is over charitable and considerate. We do not deny that Homœopathy is of utility in giving both the profession and public an opportunity to observe the progress of diseases undisturbed by perturbating remedies. We may derive from it some aid in developing our knowledge of the self-limited duration of certain diseases, and the potency of spontaneous restorative influences in the human constitution. But for this, the system is entitled to no credit. This is not what it professes to teach. If Homœopathy were true, this information would not be deducible. In order for it to be of any service to science, it must be conceded that the system is a fraudulent deception. It claims to produce its results through certain specific agencies, not simply by non-interference. Consequently, however valuable it may be in unfolding the natural history of diseases, it is not less to be despised as a system of knavish deceit, if practiced with a view to its negative results. The prudent Homœopathist will certainly not claim any consideration on the score we have mentioned. And if he were so inconsiderate, we might easily adduce as a set-off to his pretensions, the fact that while his practice affords an opportunity to study the curative resources of nature on the one hand, on the other he is compelled to witness the unsuccessful struggle in instances where judicious aid would have turned the scale in favor of the individuals whose lives depend upon the issue. One such spectacle would suffice, it would seem, for philosophical curiosity, and the fact that every member of the public will at length behold such spectacles, even through the mist of medical fanaticism, involves the inevitable destruction of this system wherever it finds a foothold. With these views, we cannot sympathise with Dr. Forbes' liberality toward Homœopathy, having shown, as he does most demonstratively, its utter absurdity; and we concur with our friend, Dr. Brainard, (who has reviewed the article in the Illinois and Indiana Medical and Surgical Journal,) that if it should really be susceptible of proof that mankind are better without, than with medicine, the proper course is to put the subject at once upon this ground, and recognise it as the principle of action, or rather non-action. Far wiser would it be to do this, than to use forbearance toward a system, which, while it depends upon the supposition that this is true, to a sufficient extent, at least, to admit of the system being temporarily popular, at the same time denies unqualifiedly the powers of unassisted nature; repudiating a principle which has always been recognised in medicine, while upon this very principle it places its sole reliance.

In thus finding fault with Dr. F. we do not distrust his intentions; and we believe his essay will be eminently useful. His object is to impress in a forcible manner the spirit of progress, and to enforce the importance of availing ourselves of every source from which light may be reflected upon the paths of scientific advancement. In conclusion he brings together a series of propositions, embodying various suggestions relative to evils to be abated, and benefits to be achieved by "*Young Physic*," in carrying forward the work of medical reformation. These propositions we subjoin, premising, that while we regard each and all deserving careful attention, they do not, by any means, develope trains of thought and action which are new to the intelligent and reflecting portion of the profession of this country.

COGITANDA—EXCOGITANDA—AGENDA.

"1. To endeavor to ascertain, much more precisely than has been done hitherto, the natural course and events of diseases, when uninterrupted by artificial interference; in other words, to attempt to establish a true Natural History of human diseases,

"2. To reconsider and study afresh the physiological and curative effects of all therapeutic agents, with a view to obtain more positive results than we now possess.

"3. To endeavor to establish, as far as is practicable, what diseases are curable and what are not; what are capable of receiving benefit from medical treatment and what are not; what treatment is the best, the safest, the most agreeable; when it is proper to administer medicine, and when to refrain from administering it; &c. &c.

"4. To endeavor to introduce a more philosophical and accurate view of the relations of remedies to the animal economy and to diseases, so as to dissociate in the minds of practitioners the notions of *post hoc* and *propter hoc*.

"The general adoption by practitioners in recording their experience, of the system known by the name of the *Numerical Method*, is essential to the attainment of the ends proposed in the preceding paragraphs, as well as in many that are to follow.

"5. To endeavor to banish from the treatment of acute and dangerous diseases, at least, the ancient axiom *melius anceps remedium quam nullum*, and to substitute in its place the safer and wiser dogma—that where we are not certain of an indication, we should give nature the best chance of doing the work herself, by leaving her operations undisturbed by those of art.

"6. To endeavor to substitute for the monstrous system of Polypharmacy now universally prevalent, one that is, at least, vastly more simple, more intelligible, more agreeable, and, it may be hoped, one more rational, more scientific, more certain, and more beneficial.

"7. To direct redoubled attention to hygiene, public and private, with the view of preventing diseases on the large scale, and individually in our sphere of practice. Here the surest and most glorious triumphs of medical science are achieving and to be achieved.

"8. To inculcate generally a milder and less energetic mode of practice,, both in acute and chronic diseases; to encourage the Expectant preferably to the Heroic system—at least where the indications of treatment are not manifest.

"9. To discountenance all active and powerful medication in the acute exanthemata and fevers of specific type, as smallpox, measles, scarlatina, typhus, &c., until we obtain some evidence that the course of these diseases can be beneficially modified by remedies.

"10. To discountenance, as much as possible, and eschew the habitual use (without any sufficient reason) of certain powerful medicines in large doses, in a multitude of different diseases, a practice now generally prevalent and fraught with the m st baneful consequences.

"This is one of the besetting sins of English practice, and originates partly in false theory, and partly in the desire to see manifest and strong effects resulting from the action of medicines. Mercury, iodine, colchicum, antimony, also purgatives in general and blood-letting, are frightfully misused in this manner.

"11. To encourage the administration of simple, feeble, or altogether powerless, non-perturbating medicines, in all cases in which drugs are prescribed *pro forma*, for the satisfaction of the patient's mind, and not with the view of producing any direct remedial effect.

"One would hardly think such a caution necessary, were it not that everyday observation proves it to be so. The system of giving and also of *taking* drugs capable of producing some obvious effect—on the sensations, at least, if not on the functions—has become so inveterate in this country, that even our *placebos* have, in the hands of our modern doctors, lost their original quality of harmlessness, and often please their patients more by being made unpleasant!

"12. To make every effort not merely to destroy the prevalent system of giving a vast quantity and variety of unnecessary and useless drugs, (to say the least of them,) but to encourage extreme simplicity in the prescription of medicines that seem to be requisite.

"Our system is here greatly and radically wrong. Our officinal formulæ are already most absurdly and mischievously complex, and our fashion is to double and redouble the existing complexities. This system is a most serious impediment in the way of ascertaining the precise and peculiar powers (if any) of the individual drugs, and thus interferes, in the most important manner, with the progress of therapeutics.

"We are aware of the arguments that are adduced in defence of medicinal combinations. We do not deny that some of these combinations are beneficial, and therefore proper; but there cannot be a question as to the enormous evils, speaking generally, resulting from them. Nothing has a greater tendency to dissociate pratical medicine from science, and to stamp it as *a trade*, than this system of pharmaceutical artifice. It takes some years of the student's life to learn the very things which are to block up his path to future knowledge. A very elegant prescriber is seldom a good physician. And no wonder. Tailors, barbers, and dancing-masters, however learned they may be in the externals of gentility, are not expected to be fine gentlemen or men of fashion.

"13. To endeavor to break through the routine habit, universally prevalent, of prescribing certain determinate remedies for certain determinate diseases or symptoms of diseases, merely because the prescriber has been taught to do so, and on no better grounds than conventional tradition.

"Even when the medicines so prescribed are innocuous, the routine proceeding impedes real knowledge by satisfying the mind, and thus producing inaction. When the drugs are potent, the crime of mischief-making is superadded to the folly of empiricism. In illustration, we need merely notice the usual reference in this country, of almost all chronic diseases accompanied with derangement of the intestinal functions, to 'affection of the liver,' and the consequent prescription of *mercury* in some of its forms. We no not hesitate to say, that this theory is as far wrong as the practice founded on it is injurious; we can hardly further enhance the amount of its divarication from the truth.

"14. To place in a more prominent point of view the great value and importance of what may be termed the physiological, hygienic, or natural system of curing diseases, especially chronic diseases, in contradistinction to the pharmaceutical or empirical drug-plan generally prevalent. This system founded as it is on a more comprehensive inquiry into *all* the remote and exciting causes of disease, and on a more thorough appreciation of *all* the discoverable disorders existing in all the organs and functions of the body, does not, of course, exclude the use of drugs, but regards them (generally speaking) as subservient to hygienic, regimenal, and external means,—such as the rigid regulation of the diet, the temperature and purity of the air, clothing, the mental and bodily exercise, &c., baths, friction, change of air, travelling, change of occupation, &c. &c.

"15. To endeavor to introduce a more comprehensive and philosophical system of Nosology, at least in chronic diseases, whereby the practitioner may be led less to consider the name of a disease, or some one symptom or some one local affection in a disease, than the disease itself—that is, *the whole* of the derangements existing in the body, and which it is his object to remove, if possible.

"16. To teach teachers to teach the rising generation of medical men, that it is infinitely more *practical* to be master of the elements of medical science, and to know diseases thoroughly, than to know by rote a farrago of receipts, or to be aware that certain doctors, of old or recent times, have said that certain medicines are good for certain diseases.

"17. Also to teach students that no systematic or theoretical classification of diseases, or of therapeutic agents ever yet promulgated, is true, or anything like the truth, and that none can be adopted as a safe guide in practice. It is, however, well that these systems should be known; as most of them involve some pathological truths, and have left some practical good behind them.

"18, To endeavor to enlighten the public as to the actual powers of medicines, with a view to reconciling them to simpler and milder plans of treatment. To teach them the great importance of having their diseases treated in their earliest stages, in order to obtain a speedy and efficient cure; and, by some modification in the relations between the patient and practitioner, to encourage and facilitate this early application for relief.

"19. To endeavor to abolish the system of medical practitioners being paid by the amount of medicine sent in to their patients; and even the practice of keeping and preparing medicines in their own houses.

"Were a proper system introduced for securing a good education to chemists and druggists, and for examining and licensing them—all of easy adoption—there could be no necessity for continuing even the latter practice; while the former is one so degrading to the medical character, and so frightfully injurious to medicine in a thousand ways, that it ought to be abolished forthwith, utterly and forever.

"20. Lastly, and above all, to bring up the medical mind to the standard necessary for studying, comprehending, appreciating, and exercising the most complex and difficult of the arts that are based on a scientific foundation,—the art of Practical Medicine. And this can only be done by elevating, in a tenfold degree, the preliminary and fundamental education of the Medical practitioner."

